# Genome-Wide Association Study Identifies Genetic Loci Associated with Iron Deficiency

**DOI:** 10.1371/journal.pone.0017390

**Published:** 2011-03-31

**Authors:** Christine E. McLaren, Chad P. Garner, Clare C. Constantine, Stela McLachlan, Chris D. Vulpe, Beverly M. Snively, Victor R. Gordeuk, Debbie A. Nickerson, James D. Cook, Catherine Leiendecker-Foster, Kenneth B. Beckman, John H. Eckfeldt, Lisa F. Barcellos, Joseph A. Murray, Paul C. Adams, Ronald T. Acton, Anthony A. Killeen, Gordon D. McLaren

**Affiliations:** 1 Department of Epidemiology, University of California Irvine, Irvine, California, United States of America; 2 Centre for Molecular, Environmental, Genetic, and Analytic Epidemiology, The University of Melbourne, Melbourne, Australia; 3 Nutritional Sciences and Toxicology, University of California, Berkeley, California, United States of America; 4 Division of Public Health Sciences, Department of Biostatistical Sciences, Wake Forest University School of Medicine, Winston-Salem, North Carolina, United States of America; 5 Department of Medicine, Howard University, Washington, D.C., United States of America; 6 Department of Genome Sciences, University of Washington, Seattle, Washington, United States of America; 7 Department of Medicine, The University of Kansas Medical Center, Kansas City, Kansas, United States of America; 8 Department of Laboratory Medicine and Pathology, University of Minnesota, Minneapolis, Minnesota, United States of America; 9 Department of Genetics, Cell Biology and Developmental Biology, University of Minnesota, Minneapolis, Minnesota, United States of America; 10 School of Public Health, University of California, Berkeley, California, United States of America; 11 Division of Gastroenterology/Hepatology, Mayo Clinic College of Medicine, Rochester, Minnesota, United States of America; 12 London Health Sciences Centre, London, Ontario, Canada; 13 Department of Microbiology, University of Alabama at Birmingham, Birmingham, Alabama, United States of America; 14 Department of Veterans Affairs Long Beach Healthcare System, Long Beach, California, United States of America; 15 Division of Hematology/Oncology, Department of Medicine, University of California Irvine, Irvine, California, United States of America; University of Frankfurt - University Hospital Frankfurt, Germany

## Abstract

The existence of multiple inherited disorders of iron metabolism in man, rodents and other vertebrates suggests genetic contributions to iron deficiency. To identify new genomic locations associated with iron deficiency, a genome-wide association study (GWAS) was performed using DNA collected from white men aged ≥25 y and women ≥50 y in the Hemochromatosis and Iron Overload Screening (HEIRS) Study with serum ferritin (SF) ≤ 12 µg/L (cases) and iron replete controls (SF>100 µg/L in men, SF>50 µg/L in women). Regression analysis was used to examine the association between case-control status (336 cases, 343 controls) and quantitative serum iron measures and 331,060 single nucleotide polymorphism (SNP) genotypes, with replication analyses performed in a sample of 71 cases and 161 controls from a population of white male and female veterans screened at a US Veterans Affairs (VA) medical center. Five SNPs identified in the GWAS met genome-wide statistical significance for association with at least one iron measure, rs2698530 on chr. 2p14; rs3811647 on chr. 3q22, a known SNP in the transferrin (*TF*) gene region; rs1800562 on chr. 6p22, the C282Y mutation in the *HFE* gene; rs7787204 on chr. 7p21; and rs987710 on chr. 22q11 (GWAS observed P<1.51×10^−7^ for all). An association between total iron binding capacity and SNP rs3811647 in the *TF* gene (GWAS observed P = 7.0×10^−9^, corrected P = 0.012) was replicated within the VA samples (observed P = 0.012). Associations with the C282Y mutation in the *HFE* gene also were replicated. The joint analysis of the HEIRS and VA samples revealed strong associations between rs2698530 on chr. 2p14 and iron status outcomes. These results confirm a previously-described *TF* polymorphism and implicate one potential new locus as a target for gene identification.

## Introduction

Iron is essential for life, but excess iron that is not safely bound to proteins can generate toxic free radicals and consequently body iron levels are tightly regulated in humans [1], [2]. Because humans do not possess an active mechanism for iron excretion, this regulation is brought about largely by modulating the uptake of iron from the diet by the enterocytes of the proximal small intestine and transfer of this iron to the systemic circulation. The same factors that regulate iron absorption also regulate release of storage iron from macrophages to the plasma to supply iron for erythropoiesis and other metabolic activities [3]. Iron deficiency is the most common nutritional disorder in the world with an estimated four to five billion affected persons [4]. Although often considered environmental in origin, the existence of multiple genetic disorders of iron metabolism in man, rodents and other vertebrates make plausible a genetic contribution to iron deficiency [5], [6], [7]. Disorders of iron metabolism underlie some of the most prevalent diseases in humans and encompass a broad spectrum of clinical manifestations, ranging from anemia to iron overload and neurodegenerative diseases [8]. Understanding the molecular basis of iron regulation in the body is critical for identifying the underlying causes of each disease entity and providing proper diagnosis and treatment [8].

We hypothesized that common variants in genes involved in iron metabolism may modulate susceptibility or resistance to the development of iron deficiency in humans. A unique multiethnic population of iron deficient individuals was identified in the Hemochromatosis and Iron Overload Screening (HEIRS) Study. In the HEIRS Study, 101,168 participants were screened with serum biochemical tests of iron status and for common mutations of the *HFE* gene [9]. As expected, participants in the HEIRS Study were identified not only with biochemical evidence of iron overload but also iron deficiency. To identify genomic locations associated with iron deficiency, we performed a genome-wide association study (GWAS) using DNA collected from white HEIRS Study participants, the largest single group identified by self-reported race/ethnicity. Case-control status and seven quantitative outcomes were examined. These included serum iron (SI), total iron-binding capacity (TIBC), unsaturated iron-binding capacity (UIBC), transferrin saturation (TfS), serum ferritin concentration (SF), serum transferrin receptor (sTfR), and body iron. Some of these traits are calculated as ratios between basal trait values; independent outcomes included SI, UIBC, SF, and sTfR. The association between outcomes and each SNP was examined. A replication study for the four SNPs showing statistical significance in the GWAS was conducted in a sample of 71 cases and 161 controls that were selected from a population of 2559 veterans attending primary care clinics at a Veterans Affairs (VA) medical center.

## Materials and Methods

### Study population and replication sample

Approval for the genome-wide association study of iron deficiency was obtained from the following: Institutional Review Board of the University of California, Irvine; Institutional Review Board of the University of California, Berkeley; Institutional Review Board of the University of Minnesota; Howard University Institutional Review Board; Institutional Review Board of the University of Alabama at Birmingham; Institutional Review Board of Kaiser Permanente Center for Health Research; Institutional Review Board of Wake Forest University Health Sciences; the University of Western Ontario Research Ethics Board for Health; and the Institutional Review Board of the Department of Veterans Affairs Long Beach Healthcare System. Written informed consent was obtained from all participants. Samples were collected by the five HEIRS Field Centers [9], [10]. Selection criteria included self-report of white or Caucasian race/ethnicity only, males at least 25 years of age and females at least 50 years. Females younger than 50 years were excluded because of pre-menopausal iron depletion from blood loss. The five Field Centers encompassed six geographic locations including Alabama, California, District of Columbia, Hawaii, and Oregon in the United States, and Ontario, Canada. Participants enrolled in the initial screening phase of the HEIRS Study were eligible if they had not withdrawn consent and agreed to blood storage. Cases had a serum ferritin concentration (SF) ≤ 12 µg/L. An equal number of iron-replete Caucasian controls (SF>100 µg/L in men, SF>50 µg/L in women) were frequency–matched 1∶1 to cases by sex and geographic location.

Replication for SNPs identified from the main GWAS was conducted in a population attending primary care clinics at a Veterans Affairs (VA) medical center, the Department of Veterans Affairs Long Beach Healthcare System. The Institutional review board reviewed and approved the study. Eligibility within the VA population was restricted to age as for the HEIRS population (≥25 y men and ≥50 y for women) and to self-reported white ethnicity. Participants were recruited by first defining a sampling frame using medical center data on patients who made outpatient visits to the facility. There were 2559 enrolled in the study (138 women). Those persons with SF≤20 µg/L, indicating low iron stores, were classified as iron-deficient cases. Milman et al. found that a serum ferritin concentration of 20 µg/L showed the highest diagnostic efficiency for identifying reduced iron stores [11]. This threshold, selected to provide an increase in power for replication, is consistent with recommendations for screening for iron deficiency in men [12]. Controls were men with SF>100 µg/L and women with SF>50 µg/L, as for GWAS participants, and they were frequency matched with cases by sex to achieve two controls for every case.

### Laboratory methods

*HFE* C282Y and H63D genotypes were determined using the Invader® Assay (Third Wave Technologies, Madison WI). Lack of a detectable C282Y or H63D mutation was designated as *HFE* wild-type (wt/wt). For the HEIRS Study, spectrophotometric measures of serum iron and UIBC levels, turbidometric immunoassay of SF (Roche Applied Science/Hitachi 911, Indianapolis, IN), and calculation of TfS were performed on non-fasting blood samples. The Central Laboratory, located at University of Minnesota Medical Center, Fairview, Minneapolis, MN, performed all laboratory tests, except TfS testing of Canadian participants. These tests were performed at MDS Laboratory Services, Canada, using an identical method. The detection threshold of the laboratory instruments for TfS was 3% and values below this detection threshold were imputed as 1.5%. The serum ferritin method was optimized to enhance the precision of measurements within the iron deficient range required for the calculation of body iron. Serum iron, sTfR, SF, and UIBC were analyzed using Roche reagents on the Roche/Hitachi Modular P instrument (Roche Diagnostics, Indianapolis, IN). TIBC was calculated as the sum of SI + UIBC. TfS was calculated as the ratio, SI/TIBC, and expressed as a percentage. Body iron (mg/kg), an index of iron deficiency, was assessed as follows: body iron  =  −[log_10_((sTfR ×1000)/SF) − 2.8229]/0.1207. In this approach, body iron is expressed as a positive value when stores are present and negatively with tissue iron deficiency [13], [14]. A body iron < −4 mg/kg body weight represents a deficit severe enough to produce anemia. However, positive values may occur in some cases of iron deficiency, for example, when sTfR is not elevated as a result of a lack of erythropoietin related to co-morbid conditions such as kidney disease. A body iron < −4 mg/kg body weight represents a deficit severe enough to produce anemia. The sTfR/SF ratio was calibrated previously by quantitative phlebotomy performed in healthy subjects [15]. To exclude common environmental causes of iron deficiency, antibody testing was performed for *H. pylori*, carcinoembryonic antigen (CEA), and celiac disease. The celiac disease screen was performed using a sequential approach. First, for all samples, the anti-tissue transglutaminase IgA (ttg) was measured. For those samples showing positive or borderline results for the ttg, an anti-endomysial antibody (ema-IgA) test was then performed. C-reactive Protein (CRP), alanine aminotransferase (ALT), and gamma-glutamyltransferase (GGT) were measured to identify acute phase protein elevations in SF.

### Genome-wide genotyping and quality control procedures for HEIRS samples

Buffy coat DNA was extracted and purified by SDS cell lysis followed by a salt precipitation method for protein removal using commercial Puregene® reagents (Gentra System, Inc., Minneapolis, MN, now Qiagen, Valencia, CA). GWAS genotyping was performed on 361 cases and 352 controls with the Illumina HumanCNV370K BeadChip platform. Fourteen cases were excluded because they had positive test results for celiac disease. Two cases and one control were excluded because they reported previous phlebotomy treatment. A total of 348,336 SNPs (excluding SNPs that are markers specifically for copy number variants) were assessed for quality. Quality control tests were carried out using the GenABEL library [16] of the R statistical package (http://www.r-project.org/). Quality control assessments resulted in the exclusion of 17,267 SNPs due to at least one of the following criteria: a minor allele frequency less than 1%, a call rate less than 95%, rejection of Hardy-Weinberg equilibrium (HWE) at p-value < 1×10^−7^ (genome-wide corrected p-value ∼0.05), or evidence for SNPs labeled as X-linked actually being autosomal at odds >1000. Eighteen samples were excluded from the analysis due to a call rate <95%, excessive genome-wide heterozygosity, an average identity-by-state value indicating a first or second degree relationship between individuals, or misclassification of sex. After filtering of SNPs and samples, there were 331,060 SNPs available for analysis in a sample of 336 cases and 343 controls. Multidimensional scaling of a matrix of identity-by-state (IBS) distances computed from the SNP genotype data found no evidence for heterogeneity in ancestry or other outliers.

### Genotyping and quality control procedures for replication samples

DNA was extracted from whole blood aliquots using an automated nucleic acid purification robotic workstation (MagNAPure, Roche) in combination with magnetic bead-based reagent technology (LC DNA Isolation kit I, Roche). The total DNA yield and quality was determined by A_260nm_ and A_280nm_ spectrophotometric readings. The four SNPs included in the replication study and presented here were included in a set of 60 SNPs that were to be genotyped on the VA samples. Multiplex design for Sequenome® MassARRAY® iPLEX Gold platform included 59 out of 60 selected SNPs divided into three multiplexes containing 32, 24 and 3 SNPs, respectively. Two big multiplexes, with 56 SNPs in total, were used for iPLEX genotyping of 238 samples. SNP call rate was >95% for 232 (97%) samples with one SNP (rs5925535) failing completely. Each sample was assessed for completeness of data and genotype data was assessed for deviations from HWE, allele frequency and completeness. The four SNPs included in the GWAS replication study passed all of the quality control assessments.

### Statistical analyses

Statistical analyses of the GWAS genotype data were carried out using the GenABEL library [16] of the R statistical package (http://www.r-project.org/). The dichotomous case-control outcome was analyzed using logistic regression and the quantitative iron status outcomes were analyzed using linear regression. Genotypes were coded as 0, 1 or 2, indicating the number of copies of the less frequent of the two alleles in the genotype. The effect of the additive genotype parameter was estimated assuming that the variable had a continuous distribution. The odds ratios reflect the multiplicative increase in risk (for being a case) attributable to the addition of one copy of the minor allele to the genotype. A positive linear regression coefficient indicates that increasing values of the quantitative outcome are associated with increasing copies of the minor allele in the genotype. The regression models for all outcomes included the additive genotype term and the covariates: age, sex and a five-level factor indicating the center where the sample was collected. Analysis of the follow-up genotype data was carried out using the SAS package with the same analytical approach as the GWAS. Significant covariates were identified by forward stepwise regression. The GWAS and follow-up combined data analysis was carried out using SAS. Data from males and females were pooled, for sample size considerations, and regression models included the additive genotype term, age, sex and a six-level factor indicating the sample source.

Missing genotypes and genotypes from unmeasured SNPs across the two regions were imputed using the program MACH 1.0 [17]. Phased haplotypes were downloaded from the HapMap database (http://www.hapmap.org) as input for the imputation. The estimated allele dose for each imputed SNP was analyzed as described for the measured SNPs. The allele dose was the product of the computed posterior probability of each genotype given the measured genotype data and the HapMap phased haplotype data and the allele dose for the genotype (0, 1 or 2 reflecting the number of minor alleles in the genotype), summed over the three possible genotypes.

## Results

### Genome-wide Association Study

Following quality control analyses, the genome-wide association analysis with 331,060 SNP genotypes was conducted on 336 iron deficient cases and 343 normal controls. The outcomes analyzed for association included the dichotomous case-control status and the seven iron-related quantitative phenotypes. Natural log transformations were applied to SF, TfS, and sTfR variables to correct for positive skewness and improve the fit to the normal distribution. Log_e_(SF) and body iron showed bimodal distributions that reflected the definition of the iron deficient case-control outcome. Table 1 shows the characteristics of the quantitative variables by case-control status for the HEIRS GWAS sample. All of the variables were significantly associated with iron deficiency case-control status (p-value<0.001).

**Table 1:** Descriptive statistics of the GWAS and replication sample phenotypes.

Measure^a^	Status	Mean	SD	Min	Max
**HEIRS GWAS Sample (336 Cases, 343 Controls)**					
Body iron	Case	−2.00	2.38	−10.04	2.40
	Control	10.82	2.50	5.71	19.31
Serum iron (µg/dL)	Case	52.5	27.80	11.0	173.0
	Control	89.65	29.32	22.0	189.0
Log_e_(SF) (µg/L)	Case	2.15	0.32	<0.01	2.57
	Control	4.96	0.61	3.95	6.73
Log_e_(TfS) (%)	Case	2.44	0.58	1.10	3.71
	Control	3.31	0.36	1.61	4.49
Log_e_(sTfR) (µg/L)	Case	1.71	0.45	0.53	3.29
	Control	1.05	0.30	0.10	2.00
TIBC (µg/dL)	Case	402.2	52.10	263.0	593.0
	Control	312.4	46.79	97.0	442.0
UIBC (µg/dL)	Case	349.7	62.76	185.0	554.0
	Control	222.7	52.89	21.0	384.0
**VA Replication Sample (71 Cases, 161 Controls)**					
Body iron	Case	−0.24	2.72	−7.88	6.01
	Control	11.04	2.78	4.74	20.9
Serum iron (µg/dL)	Case	71.4	40.6	15.0	258.0
	Control	92.4	38.7	19.0	263.0
Log_e_(SF) (µg/L)	Case	2.32	0.58	0.69	3.00
	Control	5.34	0.69	3.93	7.47
Log_e_(TfS) (%)	Case	2.80	0.60	1.13	4.26
	Control	3.26	0.40	1.93	4.51
Log_e_(sTfR) (µg/L)	Case	2.02	0.44	0.92	3.54
	Control	1.85	0.40	0.64	2.98
TIBC (µg/dL)	Case	384.5	67.20	253.7	577.6
	Control	334.2	61.1	164.2	597.1
UIBC (µg/dL)	Case	313.1	88.0	90.4	547.6
	Control	241.8	65.1	26.5	536.1

a Quantitative measure (abbreviation): serum ferritin concentration (SF), transferrin saturation (TfS), serum transferrin receptor (sTfR), total iron-binding capacity (TIBC), unsaturated iron-binding capacity (UIBC).

**Figure 1:**
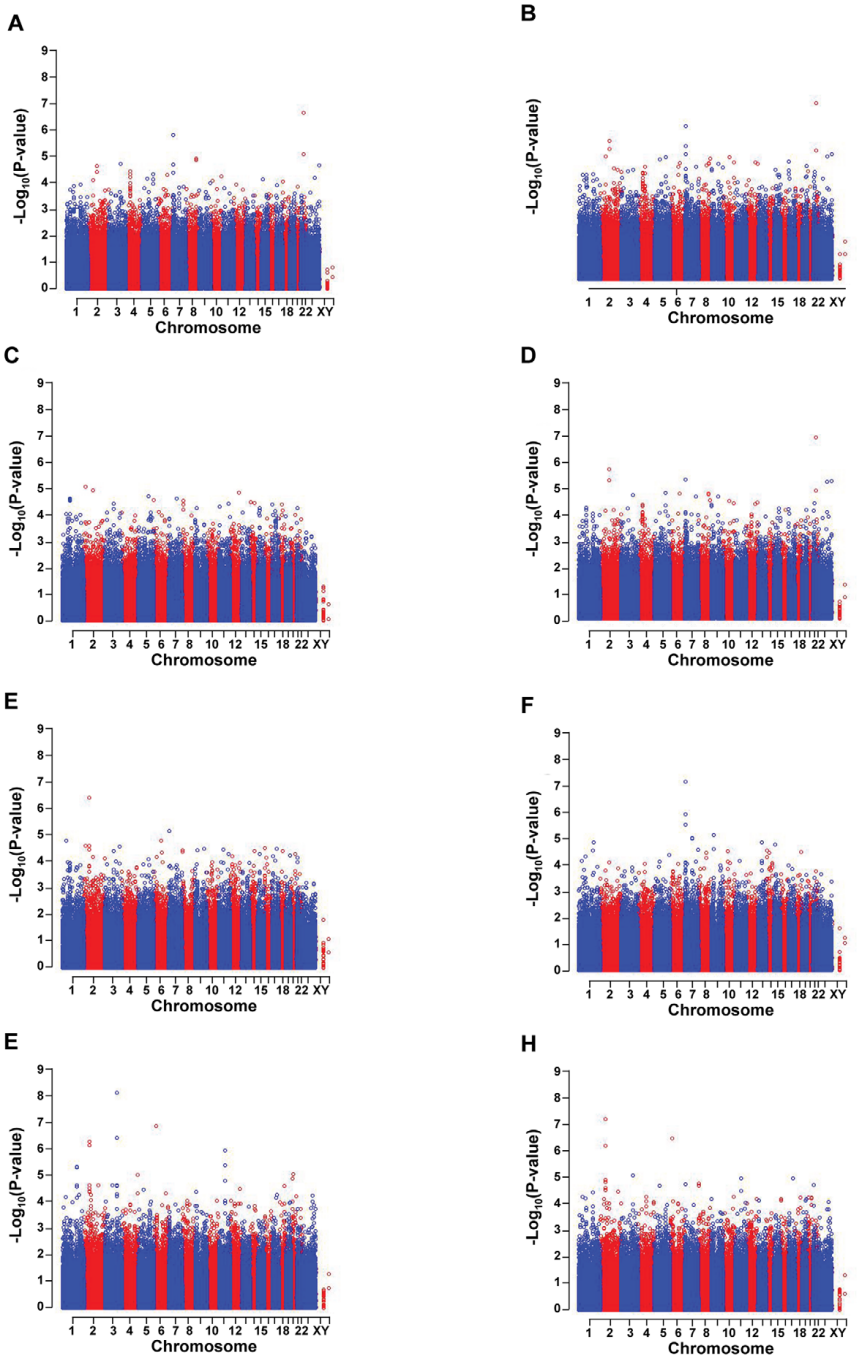
Manhattan plots displaying results from GWAS for eight iron outcomes. (A) iron deficient case-control status, (B) body iron (C) serum iron, (D) Log_e_(SF), (E) Log_e_(TfS), (F) Log_e_(sTfR), (G) TIBC, and (H) UIBC.

The full GWAS results for the eight iron status outcomes are presented graphically in the Manhattan plots shown in Figure 1. The patterns observed across the panel figures illustrate the correlations between the outcomes. Genome-wide statistical significance was defined as a SNP showing a p-value for association less than 1.51×10^−7^ for at least one of the eight iron outcomes; the threshold is based on a nominal alpha of 0.05 with Bonferroni multiple test correction for the total number of SNPs analyzed. No additional multiple test correction was made for the analysis of the correlated iron status outcomes. Table 2 shows the association results for the five SNPs meeting genome-wide statistical significance. The table shows the results for the outcome that showed the statistically significant association as well as any of the other iron status outcomes that were associated with the SNP at a p-value less than 0.01 (observed). The SNP rs2698530 on chromosome 2p14 was significantly associated with UIBC (observed p-value = 5.96×10^−8^, corrected p-value = 0.02) and approached genome-wide statistical significant associations with Log_e_(TfS) (observed p-value = 3.70×10^−7^, corrected p-value = 0.12) and TIBC (observed p-value = 5.01×10^−7^, corrected p-value = 0.17). The additional five iron outcomes showed associations with rs2698530 at p-values ranging from 2.29×10^−5^ (body iron) to 1.88×10^−4^ (serum iron).

**Table 2:** Results of genome-wide, follow-up and combined association studies.

					GWAS^b^		Follow-up Study^b^		Combined	
Chromosome,	SNP	Minor	Position	Associated					% Variance	
*Gene*		Allele		Outcome^a^	Estimate^c^	P-value	Estimate^c^	P-Value	Or O.R.	P-Value
Chr2p14	rs2698530	C	64357399	Case-Control	1.68	7.86×10^−5^	0.85	0.52	1.44 (O.R.)	0.0014
				Body Iron	−1.81	2.29×10^−5^	−0.20	0.76	1.5	0.00019
				Serum Iron	−7.94	1.88×10^−4^	2.02	0.66	0.7	0.0083
				Log_e_(SF)	−0.36	8.23×10^−5^	0.03	0.85	1.0	0.0018
				Log_e_(TfS)	−0.20	3.70×10^−7^	−0.02	0.71	2.3	3.51×10^−6^
				Log_e_(sTfR)	0.12	1.63×10^−4^	0.12	0.013	1.6	4.00×10^−5^
				TIBC	20.72	5.01×10^−7^	16.27	0.032	3.0	1.67×10^−7^
				UIBC	28.75	5.96×10^−8^	14.25	0.11	3.0	1.40×10^−7^
Chr3q22.1, *TF*	rs3811647	A	134966719	Case-Control	1.07	0.58	0.91	0.65	1.08 (O.R.)	0.44
				Body Iron	−0.28	0.48	0.16	0.78	0.03	0.60
				Serum Iron	−0.19	0.92	7.88	0.045	0.11	0.31
				Log_e_(SF)	−0.06	0.48	−0.04	0.79	0.07	0.42
				Log_e_(TfS)	−0.06	0.088	0.05	0.32	0.15	0.24
				Log_e_(sTfR)	0.02	0.58	−0.07	0.096	0.02	0.68
				TIBC	21.49	7.00×10^−9^	16.69	0.012	4.2	4.50×10^−10^
				UIBC	21.62	7.78×10^−6^	8.81	0.26	2.2	8.35×10^−6^
Chr6p22.2, *HFE*	rs1800562	A	26093141	Case-Control	0.76	0.19	0.68	0.24	0.89	0.53
				Body Iron	1.43	0.042	0.75	0.41	0.55	0.022
				Serum Iron	9.07	0.0091	21.16	0.0005	1.9	1.93×10^−5^
				Log_e_(SF)	0.31	0.039	0.06	0.81	0.41	0.047
				Log_e_(TfS)	0.19	0.005	0.22	0.0066	1.5	0.00016
				Log_e_(sTfR)	−0.08	0.12	−0.17	0.0072	0.31	0.074
				TIBC	−35.36	1.31×10^−7^	−21.58	0.035	3.4	2.68×10^−8^
				UIBC	−44.27	3.15×10^−7^	−42.7	0.0004	4.1	5.72×10^−10^
Chr7p21.3	rs7787204	G	9847296	Case-Control	2.25	3.94×10^−5^	1.01	0.98	1.86 (O.R.)	0.00015
				Body Iron	−2.72	8.93×10^−6^	0.21	0.82	1.8	4.24×10^−5^
				Serum Iron	−6.99	0.023	−1.08	0.86	0.50	0.03
				Log_e_(SF)	−0.52	9.29×10^−5^	0.06	0.80	1.2	0.00075
				Log_e_(TfS)	−0.18	0.0016	−0.05	0.50	1.2	0.0011
				Log_e_(sTfR)	0.24	7.30×10^−8^	−0.02	0.79	0.7	0.0071
				TIBC	19.44	0.0011	13.45	0.18	1.4	0.00029
				UIBC	26.46	5.83×10^−4^	14.53	0.22	1.6	0.00015
Chr22q11.22	rs987710	G	22512415	Case-Control	0.54	2.13×10^−7^	0.85	0.48	0.60 (0.R.)	1.28×10^−6^
				Body Iron	2.01	2.21×10^−7^	0.76	0.20	2.7	5.23×10^−7^
				Serum Iron	5.68	0.0036	3.76	0.35	0.8	0.0069
				Log_e_(SF)	0.44	1.40×10^−7^	0.19	0.22	2.5	1.16×10^−6^
				Log_e_(TfS)	0.12	0.0018	0.10	0.043	1.3	0.00044
				Log_e_(sTfR)	−0.11	1.73×10^−4^	−0.05	0.26	0.6	0.011
				TIBC	−10.22	0.0073	−8.33	0.20	0.8	0.0059
				UIBC	−15.93	0.0012	−12.08	0.12	1.2	0.00088

a Quantitative measure (abbreviation): serum ferritin concentration (SF), transferrin saturation (TfS), serum transferrin receptor (sTfR), total iron-binding capacity (TIBC), unsaturated iron-binding capacity (UIBC).

b For analysis of the GWAS genotype data, the regression models for all outcomes included the additive genotype term and the covariates, age, sex and a five-level factor indicating the center where the sample was collected. For analysis of the follow-up genotype data, the regression models for all outcomes included the additive genotype term and the covariates, age and sex.

c For case-control analyses only, the odds ratios estimate the odds in favor of being iron deficient (over being iron replete) among those participants who have one additional minor allele, divided by the odds of being iron deficient among those without the additional minor allele. For all other outcomes, a positive linear regression coefficient indicates that increasing values of the quantitative outcome are associated with increasing copies of the minor allele in the genotype.

The SNP rs3811647 in the *TF* gene on chromosome 3q22.1 showed a significant association with TIBC (observed p-value = 7.00×10^−9^, corrected p-value = 0.0023). The SNP also showed an observed p-value of 7.78×10^−5^ for association with UIBC that did not meet genome-wide statistical significance and there was no evidence for association with any of the six other iron status outcomes. The SNP rs7787204 on chromosome 7p21.3 showed a statistically significant association with Log_e_(sTfR) (observed p-value = 7.30×10^−8^, corrected p-value = 0.024). For this SNP, six of the other iron outcomes had observed p-values of less than 0.01, with body iron (p-value = 8.93×10^−6^) and iron deficient case-control status (p-value = 3.49×10^−5^) showing the smallest observed p-values of the six outcomes. The SNP rs987710 on chromosome 22q11.22 showed a significant association with Log_e_(SF) (observed p-value = 1.40×10^−7^, corrected p-value = 0.046) and nearly met the threshold for genome-wide significance with iron deficient case-control status (observed p-value = 2.13×10^−7^, corrected p-value = 0.071) and body iron (observed p-value = 2.21×10^−7^, corrected p-value = 0.073). The five remaining iron outcomes showed marginal evidence for association with the SNP.

The SNP rs1800562 on chromosome 6p22.2 is the C282Y mutation in the *HFE* gene and is known to affect iron metabolism. The SNP met genome-wide statistical significance for association with TIBC (observed p-value = 1.31×10^−7^, corrected p-value = 0.043) and nearly reached genome-wide statistical significance for UIBC (observed p-value = 3.15×10^−7^, corrected p-value = 0.10), and showed marginal evidence for association with serum iron (observed p-value = 0.0091) and Log_e_(TfS) (observed p-value = 0.005). The known iron overload mutation did not show statistically significant evidence for association with iron deficient case-control status, body iron, Log_e_(SF) and Log_e_(sTfR), even at a nominal alpha level of 0.01. The allele frequency of the mutation in the full GWAS sample was 0.074 with case and control frequencies of 0.063 and 0.085 respectively.

The genomic control parameters (i.e., lambda) computed from the case-control association statistics was 0.996 (SE = 0.00002) indicating that no genome-wide inflation of the association statistics was present. Genomic control parameters were also computed for the log_e_(SF) and body tissue iron outcomes because the variables showed bimodal sample distributions which would result in a violation of the assumption of normally distributed residual errors in the linear regression analysis and the potential for an incorrect false positive rate. Neither of the two outcomes showed evidence for a significant deviation from the expected null distribution with both having genomic control parameters of 1.002 (SE<0.0001).

### Follow-up Association Study

A follow-up sample of 71 white iron deficient cases (61 males, 10 females) and 161 matched controls (134 males, 27 females) from the Department of Veterans Affairs Long Beach Healthcare System was used to assess replication of the GWAS findings. Table 1 shows the characteristics of the quantitative iron outcomes by case-control status. All variables were significantly associated with case-control status (p<0.001). Table 2 shows the results of the association analysis of the follow-up sample for the four SNPs that showed significant associations in the GWAS as well as the C282Y mutation. The SNPs in the follow-up analysis were tested for association with statistical models that included age and sex so the results would be comparable to the GWAS. Using the SF threshold of 20 for case definition may have introduced more heterogeneity in samples and possibly increased the false negative rate for detection of association with iron measures; however, the criteria for replication of association was still achieved for several SNPs. The follow-up analysis of rs2698530 (chr. 2p14), rs3811647 (*TF*) and rs1800562 (*HFE*) showed evidence for association with an observed p-value less than 0.05 with an iron outcome that met or nearly met the genome-wide significance level in the GWAS. The SNP rs2698530 showed observed p-values of 5.01×10^−7^ and 0.032 for association with TIBC in the GWAS and follow-up analyses, respectively. P-values of 7.0×10^−9^ and 0.012 were observed for the association between rs3811647 and TIBC in the GWAS and follow-up studies, respectively. UIBC and TIBC both showed evidence for association with the C282Y mutation in the follow-up study, with p-values of 0.0004 and 0.035, respectively. The SNPs rs7787204 on chromosome 7p21.3 and rs987710 on 22q11.22 did not show any evidence for association and failed to replicate in the follow-up sample.

### Association Analysis of Combined GWAS and Follow-up Samples

The GWAS and follow-up study data were combined and the five SNPs shown in Table 2 were analyzed for each of the outcomes in a regression model that included age, sex and the collection center variable. The results of the combined analysis are consistent with the follow-up study. Genome-wide significance was found for the association of rs2698530 (chr. 2p14) with UIBC (observed p-value = 1.40×10^−7^, corrected p-value = 0.046) and statistical significance was nearly met with TIBC (observed p-value = 1.67×10^−7^, corrected p-value = 0.055). Significant association between rs3811647 in the *TF* gene and TIBC was found in the combined sample (observed p-value = 4.50×10^−10^, corrected p-value = 0.00015). The C282Y mutation in the *HFE* gene showed significant associations with UIBC (observed p-value = 5.72×10^−10^, corrected p-value = 0.00019) and TIBC (observed p-value = 2.68×10^−8^, corrected p-value = 0.0089) in the combined sample.

### High-Resolution Association Analysis of Chromosomes 2p14 and 3q22

The strongest statistical evidence for association was found at SNPs on chromosomes 2p14 and 3q22. In order to map the associations within the two regions with higher resolution, genotypes from sets of measured SNPs across the two regions were used to impute unmeasured genotypes so that a higher density of SNPs could be analyzed. The significantly associated SNPs in both regions were observed within single blocks of high linkage disequilibrium (LD) that were bounded by recombination hotspots. The high-resolution analysis included the complete blocks of high linkage disequilibrium where the significantly associated SNPs were located and extended tens of kbp into the neighboring blocks on each side. For chromosome 2, the fine resolution analysis extended over approximately 128 kbp. The high-resolution analysis of chromosome 3 included a region of approximately 279 kbp; however, the results are presented for 32.5 kbp of this region in order to better illustrate the important results. The genomic positions are based on the March 2008 human reference sequence (NCBI Build 36.3).

**Figure 2:**
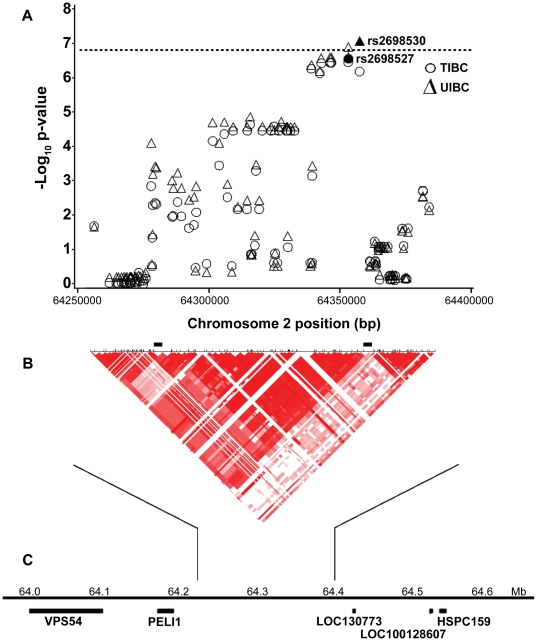
High resolution association analysis of GWAS samples for chromosome 2p14. Analysis includes measured genotypes from 21 SNPs and imputed genotypes from 108 SNPs. A. Genome-wide statistical significance is represented by the dashed line corresponding to an observed p-value of 1.51×10^−7^. For unsaturated iron-binding capacity (UIBC), the most significant association was with rs2698530 (▴) with an observed p-value = 8.31×10^−8^. For total iron-binding capacity (TIBC), the most significant association was with rs2698527 (•) with an observed p-value = 2.73×10^−7^. B. The heat graph was generated from pairwise LD coefficients D', calculated from the HapMap genotype data for all 129 SNPs. Recombination hotspots are indicated by black bars. C. The location of the region on chromosome 2p14, with approximate position and size of neary genes is shown.

Figure 2 shows the results of the high-resolution association analysis of TIBC and UIBC across the 127,705 bp region of chromosome 2p14. The analysis included 117 SNPs; 21 SNPs were measured and analyzed in the GWAS and the remaining 96 SNPs were generated using imputation. The most significant results were found at SNPs that were part of the GWAS. For UIBC the most significant association was at rs2698530 (observed p-value = 8.31×10^−8^) and for TIBC it was at rs2698527 (observed p-value = 2.73×10^−7^). A continuous set of eight SNPs showed –log_10_(p-values) greater than 6.0 (i.e., p-value<1.0×10^−6^) for association with both UIBC and TIBC, spanning 15,216 bp, bounded by rs2698541 and rs2698530. The region contains no known genes. Pair-wise D' LD measures were computed from all of the SNPs included in the high-resolution association analysis and were presented in the two-dimenional graph shown under the association results. The graph clearly shows that the LD block boundaries are consistent with the association results with steep drops in the statistical significance of the association results occurring at the boundaries of the high-LD block.

**Figure 3:**
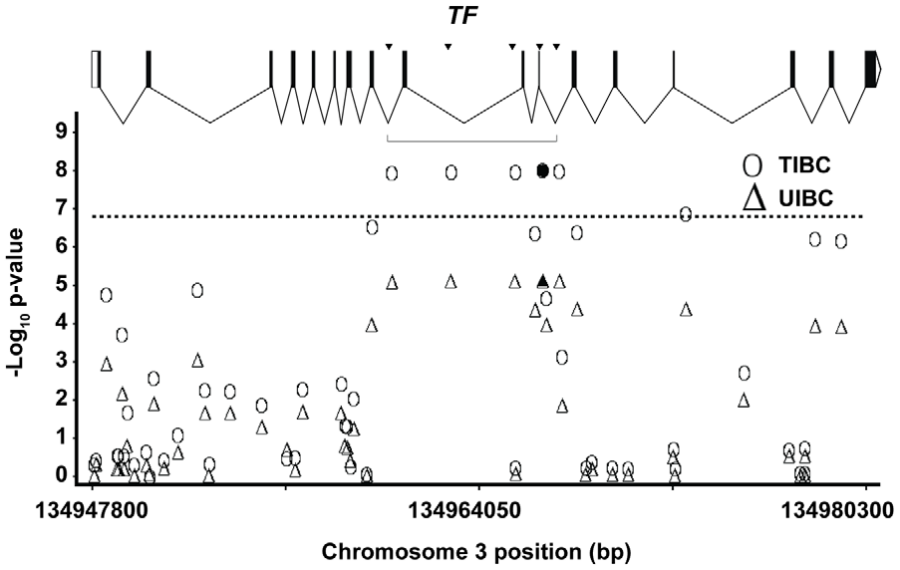
High resolution association analysis of GWAS samples for chromosome 3q22. The analysis includes measured genotypes from 40 SNPs and imputed genotypes from 220 SNPs. The location and structure of the *TF* gene is shown along the top of the figure. The most significant associations with total iron-binding capacity (TIBC, •) and unsaturated iron-binding capacity (UIBC, ▴) were observed with the measured SNP rs3811647, with observed p-values of 9.55×10^−9^ and 7.28×10^−6^ for TIBC and UIBC, respectively. The most statistically significant associations were observed at five SNPs across the 7 kbp region (delineated) which includes exons 9, 10, and 11 of the transferrin gene, *TF*.

The high-resolution association analysis of chromosome 3q22 included 40 SNPs measured in the GWAS and 220 imputed SNPs distributed across a 279,147 bp region. Figure 3 shows the results from the analysis of TIBC and UIBC across 32,500 bp of the full region analyzed. The location and structure of the *TF* gene is shown across the top of the figure. The most significant association with both outcomes was observed with the measured SNP rs3811647, with observed p-values of 9.55×10^−9^ and 7.28×10^−6^ for TIBC and UIBC, respectively. The most statistically significant associations with TIBC and UIBC were observed at five SNPs across a 7,011 bp region. The region included one measured SNP (rs3811647) and four imputed SNPs (rs8177240, rs8177252, rs8177272 and rs1525892). The 7 kbp region includes exons 9, 10 and 11 of the *TF* gene.

## Discussion

In this genome-wide association study of participants in the HEIRS Study, analyses of data from iron-deficient cases and iron-replete controls identified five SNPs associated with at least one of the quantitative iron measures at an observed p-value less than 1.51×10^−7^ (genome-wide corrected p-value<0.05). These SNPs included rs2698530 on chr. 2p14, rs3811647 in the *TF* gene on chr. 3q22, rs1800562 on chr. 6p22 (the known C282Y mutation in *HFE*), rs7787204 on chr. 7p21, and rs987710 on chr. 22q11. Replication of the association with TIBC in an independent VA population was demonstrated for rs3811647 (observed P = 0.012) and for rs1800562 in *HFE* (observed P = 0.035). In the joint analysis, combining HEIRS and VA datasets, the strongest statistical significance with iron-related measures was found at rs3811647 in the *TF* gene for association with TIBC (observed P = 4.50×10^−10^). The joint analyses also revealed strong associations between rs2698530 on chr. 2p14 and two iron status outcomes, TIBC and UIBC. High-resolution association analyses of TIBC and UIBC across a 127,705 bp region of 2p14 containing rs2698530 and a 279,147 bp region of 3q22 containing rs3811647 indicated that the most significant results were found at measured SNPs in the GWAS for association with UIBC (rs2698530, observed P = 8.31×10^−8^ Fig. 2) and with TIBC (rs3811647, observed P = 9.55×10^−9^, Fig. 3).

The quantitative iron-related measures examined here were significantly associated with iron deficiency in the GWAS (p<0.001) and in the VA follow-up sample (p<0.001), and a high degree of concordance was observed in the results across the quantitative traits. This was expected as TfS is calculated from the measured values of serum iron and UIBC, and body iron is estimated from sTfR and SF. Total iron binding capacity measures the blood's capacity to bind iron with transferrin, and values are expected to be higher in individuals with iron deficiency, compared to those who are iron replete [18]. This relationship was borne out in the current study, with higher TIBC values in the cases than in the controls. In the GWAS and follow-up studies, we used TIBC as a marker for transferrin. For purposes of comparing serum transferrin levels, TIBC and transferrin concentration may be used interchangeably. The parameter estimate was positive for rs3811647 (chr. 3q22) in the regression on TIBC, indicating that increasing values of TIBC are associated with increasing copies of the minor allele in the rs3811647 genotype. In contrast, for the regression of rs987710 (chr. 22q11) on TIBC, the parameter estimate was negative, indicating that decreasing values of TIBC are associated with increasing copies of the minor allele.

In humans, strong evidence supporting the presence of genetic modifiers of iron metabolism was reported by Whitfield and colleagues who studied a sample of both monozygotic and dizygotic twin pairs and showed that the pattern of residual variation in serum iron indices, after adjusting for an effect of the C282Y mutation, was consistent with the additive effects of multiple genes [19]. After correcting for age and body-mass index they estimated that the proportion of variance explained by additive genetic factors, for men and women respectively, Genetic variants in *TF* have previously been described and investigated in terms of association with iron status [20], [21], [22], [23], [24]. A study by Milet and colleagues of a cohort of 592 unrelated C282Y homozygous probands who attended the Liver Unit in Rennes, France, was the first to show strong evidence for an association between a measured common genetic variant, a SNP in the *BMP2* gene, and the serum ferritin levels of C282Y homozygotes [25]. *BMP6* has recently been shown to be the key endogenous regulator of hepcidin [26], [27], [28], [29]. Mutations in the *TMPRSS6* gene, another upstream regulator of hepcidin, have been implicated in iron-refractory iron deficiency anemia through linkage studies [30], [31], [32], although these results are based on a few extended pedigrees and may have limited relevance at the population level. Mutations in many other genes are known to cause serious disruption of normal iron metabolism (e.g. *HFE2*, *HAMP*, *SLC40A1, TFR2*) but the causal mutations are very rare [33]. Constantine *et al* reported an association between SNP rs884409 in *CYBRD1* and serum ferritin levels measured in *HFE* C282Y homozygotes [34]. In our study, this SNP was not available for assessment in the GWAS as it was not included on the HumanCNV370K BeadChip.

Further evidence of genetic influences on iron status was found in a recent study that investigated genetic effects on markers of iron status using a cohort of twins and their siblings. A GWAS was conducted on four serum markers of iron status (serum iron, transferrin, transferrin saturation and serum ferritin) [35]. Along with confirming previously reported associations of *HFE* C282Y on all four markers, these investigators found strong associations between serum iron and a *TMPRSS6* SNP (rs4820268), and between serum transferrin and several *TF* SNPs (rs3811647, rs1358024, rs452586) [35]. The SNP rs452586 was not in our GWAS dataset. In our study, we found a significant association between TIBC and rs3811647, but no significant association was found with rs1358024. We examined linkage disequilibrium between rs3811647 and rs1358024. The *D*' estimate of 1.0 for this SNP pair indicates very strong linkage disequilibrium, however the *r^2^* estimate of 0.39 indicates that the SNPs poorly predict their corresponding genotypes and respective associations with the iron phenotypes. An association between SNPs in *TMPRSS6* and serum iron (rs855791) and transferrin saturation has been found in adolescent and adult individuals [36]. Variants in *TMPRSS6* also have been associated with hemoglobin levels in individuals of both European and Indian Asian ancestry [37]. Tanaka and colleagues investigated genetic variants associated with iron concentrations in persons not affected by overt genetic disorders of iron metabolism. They conducted a GWAS and confirmed that rs855791 on exon 17 of *TMPRSS6* and rs4820268 on exon 13 were strongly associated with lower serum iron concentration, lower mean corpuscular volume, lower hemoglobin levels, and higher red blood cell distribution width [38]. In contrast, we found no genome-wide statistically significant associations between *TRMPRSS6* SNPs and iron-related traits in the current study. This may be related to our study design that selected iron-deficient and control groups. Use of a group that represents one extreme of the population may have masked the effect of *TMPRSS6* SNPs as reflected in a shift in the distribution of iron status measures of a large number of participants in a general population study. As complete blood count values were not collected in the screening phase of the HEIRS Study, we did not examine associations between SNPs and erythrocyte parameters.

It has been shown that heterozygosity or homozygosity for the C282Y variant of the *HFE* gene protects against the development of iron deficiency [39], [40], [41], [42]. In the present study, we examined the associations between iron-related measures and SNP rs1800562, the C282Y mutation of the *HFE* gene. Illustrating a protective effect, increasing copies of the minor allele in the C282Y genotype were associated with decreasing values of TIBC and UIBC in the GWAS (observed P<3.15×10^−7^ for both), the VA follow-up study (observed P<0.04 for both), and the combined sample (observed P<3.0×10^−9^ for both). The C282Y genotype was estimated to account for 2.0% and 3.4% of the variance in the TIBC and UIBC traits, respectively.

A limitation to this GWAS may be the relatively small number of cases with iron deficiency determined through screening of 101,168 adults. Nevertheless, four SNPs were identified that met criteria for genome-wide significance for association with iron-related measures. Of these, replication on the basis of analyses with adjustment for age and sex was achieved for the positive association between TIBC and the number of copies of the less frequent allele for SNP rs3811647, located on chr. 3q22, and a strong negative association with increasing number of copies of the less frequent allele of the C282Y mutation of the *HFE* gene. For the replication studies, a threshold of SF≤20 µg/L was used to classify cases of iron-deficiency. This clinically relevant threshold for men was selected to provide an increase in power for replication. It is possible that this may have introduced more heterogeneity in samples and may have resulted in an increase in the false negative rate for detection of association with iron measures. However, even though this conservative approach was taken, the criteria for replication of association were achieved for some SNPs.

Another limitation may be that it is unknown how likely genetic variants are to affect the variance of the measures used for determining iron status rather than the actual total body iron. Additionally, serum ferritin concentration, used to determine case status, is known to be correlated with other quantitative traits assessed in the study. It is possible that this, along with the selective genotyping of individuals from the high and low phenotypic tails of the populations, may have introduced some bias. For example, Darvasi and Soller demonstrated in linkage analyses that the observed difference in quantitative trait values associated with alternative marker genotypes in a selected population can be greater than the actual gene effect at the quantitative trait locus when the entire population is considered [43]. However, the case-control design of the current study offered the opportunity to examine specifically the question whether the *TF* gene SNP rs3811647 on Chr. 3q22 has an effect on iron status and our results suggest that it does not.

Our study design differs from previously reported studies in that the GWAS was conducted on samples from iron-deficient and control groups identified through population-based screening of participants. In addition, serum specimens from both sources were tested to exclude common environmental causes of iron deficiency and causes of acute phase protein elevations in serum ferritin. A key finding in the current study is that, although the rs3811647 SNP in *TF* was associated with TIBC (and, to a lesser extent, UIBC), it was not associated with other measures of body iron status or case-control status. These results do not support the concept of a role for this *TF* SNP in regulation of iron metabolism. Thus, this SNP may instead affect TIBC independently of iron status. Use of TIBC as an index of iron deficiency may be confounded by the existence in the population of the rs3811647 minor allele, resulting in an elevated TIBC without a corresponding increase in body storage iron. With respect to the rs1800562 SNP in *HFE* on Chr 6p22.2, we note that the four associated iron measures are serum iron, transferrin saturation, TIBC, and UIBC. It is recognized that mean levels of transferrin saturation (which is calculated from the ratio of serum iron to TIBC or the ratio of serum iron to the sum of serum iron and UIBC) are elevated in individuals with one copy of this SNP [39], [44]. However these individuals rarely have any clinically significant increase in body iron stores [39], so the lack of association with other markers of body iron status such as measurements of serum ferritin and serum transferrin receptor is not surprising. It is only individuals who are homozygous for SNP rs1800562 on chromosome 6p22.2, the C282Y mutation in the *HFE* gene, in which an increase in iron stores is likely to occur. In the HEIRS cohort there were only two cases and four controls who were homoyzygous for the C282Y mutation. The VA cohort contained one case and four controls who were C282Y homozygotes.

In summary, this study confirmed the previously identified rs3811647 in the *TF* gene on chr. 3q22 and rs1800562, the C282Y mutation in the *HFE* gene. Genome-wide association with UIBC was demonstrated for one new locus identified in the GWAS, rs269853 on chr. 2p14; however evidence of replication was marginal in the analysis of an independent VA population.
